# Editorial: The influence of bioactive compounds on metabolic syndrome

**DOI:** 10.3389/fnut.2025.1703963

**Published:** 2025-10-03

**Authors:** Yoo Kim, Bonggi Lee, Byungyong Ahn

**Affiliations:** ^1^Department of Nutritional Sciences, Oklahoma State University, Stillwater, OK, United States; ^2^Department of Food Science and Nutrition, Pukyong National University, Busan, Republic of Korea; ^3^Interdisciplinary Program of Blue Food, Pukyong National University, Busan, Republic of Korea; ^4^Department of Food Science and Nutrition, University of Ulsan, Ulsan, Republic of Korea; ^5^Basic-Clinical Convergence Research Institute, University of Ulsan, Ulsan, Republic of Korea

**Keywords:** metabolic syndrome (MetS), bioactive compounds, gut microbiota, dietary strategies, functional foods

Metabolic syndrome (MetS) is widely recognized as a major public health challenge, not only in Western societies but increasingly in Asia and other regions undergoing rapid lifestyle transitions (1, 2). MetS is not a single disease, but rather a cluster of interrelated metabolic abnormalities, including abdominal obesity, elevated blood glucose or insulin resistance, dyslipidemia, and hypertension. The coexistence of these conditions substantially heightens the risk of developing type 2 diabetes mellitus, cardiovascular disease, and other chronic health disorders. Despite the development of numerous pharmaceutical agents to manage MetS, their therapeutic effects are often limited. Most medications target individual components of the syndrome rather than addressing the condition as a whole, resulting in incomplete metabolic control and potential side effects. This limitation has prompted growing interest in alternative and complementary strategies. Among these, bioactive compounds derived from foods have attracted considerable scientific and clinical attention. In contrast to conventional drugs that act on a single pathway, these naturally occurring compounds can simultaneously influence multiple metabolic pathways. Bioactive food components may offer a more holistic, safe, and sustainable approach to the prevention and management of MetS across diverse populations by enhancing glucose and lipid metabolism, reducing inflammation, and supporting vascular health (3).

The purpose of this Research Topic was to collect studies investigating how such compounds influence MetS. The papers are not all the same type: some focus on food processing, others utilize animal models, while one analyzes population data and another reports the findings from clinical trials. Despite these methodological differences, they all share the same objective: to determine whether natural food compounds can help to prevent or manage MetS.

Lee et al. investigated the effects of food processing on enhanced functionality. Specifically, they studied parsnip (*Pastinaca sativa* L.) and assessed how post-harvest aging influenced the concentration of bioactive compounds and antioxidant activity. During aging, the phenolic content notably increased, and antioxidant activity became stronger. Compounds such as falcarindiol and 5-hydroxymethylfurfural were found in higher amounts in aged parsnip. Extracts from aged parsnip protected both cells and mice against oxidative stress and inflammation caused by acrolein. These findings are noteworthy, as even a simple process such as aging can enhance the bioactivity of an ordinary vegetable, underscoring its potential value in functional foods (Lee et al.).

Zuo et al. studied the effects of short peptide–based enteral nutrition in a septic mice model. After 1 week, mice given short peptides exhibited a stronger intestinal barrier, reduced tissue injury, and enhanced recovery of body weight. Gut microbial composition was also changed, with an increase in beneficial *Lactobacillus* and a decrease in harmful fatty acids such as dodecanoic acid and 10-heptadecenoic acid. Metabolomic analysis confirmed this result. It demonstrates again that the gut serves as a central place where nutrition can influence metabolism. In fact, this part is becoming clearer every year: the gut is not merely a site of digestion, but also a key player in systemic metabolic health (Zuo et al.).

Hou et al. tested lipid-related indices as potential biomarkers of metabolic risk, focusing on the non-HDL to HDL cholesterol ratio (NHHR) in the CHARLS cohort. More than 4,600 participants were followed for 4 years, those with higher NHHR values were more likely to develop hyperuricemia. The relationship was linear across the observed range, rather than displaying only a threshold effect. Interestingly, the association appeared stronger in women, non-smokers, and older adults. Because NHHR can be calculated easily from basic blood tests, it may be a practical marker for identifying individuals at elevated metabolic risk (Hou et al.).

Balbuena et al. investigated whether carotenoids in orange carrots could protect against high-fat diet-induced intestinal dysfunction. Carotenoid analysis confirmed that only the orange carrot diet contained provitamin A carotenoids, mainly α- and β-carotene. Quantitative proteomics of distal colon tissue identified 4,410 differentially expressed proteins. Orange carrot supplementation notably upregulated intestinal barrier–associated proteins, including mucin-2 (MUC-2), and enhanced pathways involved in mucus synthesis and secretion. Functional analyses revealed improvements in colonic epithelial protection without adverse effects on body weight. These findings suggest that carotenoid-rich vegetables can strengthen the intestinal barrier, reduce inflammation, and counteract obesity-associated colonic dysfunction (Balbuena et al.).

Liu et al. conducted a systematic review and meta-analysis of randomized controlled trials evaluating berberine, an isoquinoline alkaloid that has been widely used in Asian medicine for centuries. The review covered 12 clinical trials with approximately 900 participants. The results showed that berberine significantly lowered triglyceride levels, fasting glucose, waist circumference, LDL cholesterol, and total cholesterol. It also improved oral glucose tolerance and BMI in some trials. Importantly, berberine was generally well-tolerated, with only mild gastrointestinal discomfort such as stomach upset or constipation. The conclusion is that berberine can improve several parts of MetS at the same time and may be considered a safe complementary therapy. This is a good example of how a traditional compound can remain highly relevant when rigorously evaluated by modern clinical approaches (Liu et al.).

Looking across these studies, some common points stand out. Food processing not only keeps food fresh, but also can increase its health benefits. Parsnip is just one example of how simple changes can bring new value. The gut appears again as a very central target, and short peptides highlight this clearly. Simple combined markers such as NHHR may sometimes predict risk better than single lipid measures. Carotenoids from orange carrots protect against high-fat diet–induced colonic dysfunction. Long-used compounds like berberine remain important today. Together, these contributions provide both mechanistic insight and clinical meaning. More importantly, they remind us that nutrition and medicine are not separate fields. Many of these compounds are inexpensive, widely available, and already part of daily diets. This means they can be used not only in laboratories or clinics, but also in public health strategies, particularly in regions such as Asia where MetS is rapidly increasing.
